# Supplementary material for: Fecal fungal microbiota alterations associated with clinical phenotypes in Crohn’s disease in southwest China
Source: PeerJ. 2022 Oct 18;10:e14260. doi: 10.7717/peerj.14260 (PMC9586077; doi:10.7717/peerj.14260)
Supplement: Supplemental Information 1 [file peerj-10-14260-s001.docx]

**supplementary materials:**

Supplementary Table 1 Additional information of study population

|  | **CD all(n=45)** | **CD-flare(n=34)** | **CD-remission(n=11)** | ***P* value** | **HC（n=17）** |
| --- | --- | --- | --- | --- | --- |
| **Age, Mean±SD** | 32.0±11.6 | 32.9±11.6 | 29.4±11.7 | 0.39 | 27.6±9.7 |
| **Male, n** | 34（75.6%） | 25(73.5%) | 9(81.8%) | 0.705 | 8（47.1%） |
| **Extra-intestinal manifestations, n** |  |  |  |  |  |
| **Oral aphtha** | 6 | 5 | 1 | 1.00 | - |
| **Genital ulcer** | 2 | 2 | 0 | 1.00 | - |
| **Skin lesion** | 1 | 1 | 0 | 1.00 | - |
| **Arthritis/arthralgia** | 3 | 3 | 0 | 0.57 | - |
| **Others** | 1 | 1 | 0 | 1.00 | - |
| **Complications, n** |  |  |  |  |  |
| **abdominal abscess** | 9 | 9 | 0 | 0.09 | - |
| **Fistulas and/or sinus** | 24 | 22 | 2 | 0.01 | - |
| **Perianal lesion** | 25 | 20 | 5 | 0.50 | - |
| **Treatment, n** |  |  |  |  |  |
| **Sulfasalazine** | 4* | 3* | 1 | 1.00 | - |
| **Corticosteroids** | 3 | 3 | 0 | 0.57 | - |
| **Thiopurine or Methotrexate** | 17# | 16 | 1# | 0.03 | - |
| **anti-TNFα** | 16$ | 9 | 7$ | 0.04 | - |
| **None** | 6 | 3 | 3 | 0.15 | - |

CD-flare, CD at active stage (i.e. CDAI score ≥150); CD-remission, CD at remission stage (i.e. CDAI score <150); * one patient treated with sulfasalazine and etanercept; # one patient treated with AZA and IFX.

**Supplementary Table 2**  Alpha diversity between CD group and HC group

| **Group** | **observed species** | **Shannon index** | **PD-whole tree** |
| --- | --- | --- | --- |
| **CD** | 66.6±19.6 | 2.8±1.3 | 14.3±3.6 |
| **HC** | 125.4±212.0 | 3.0±1.7 | 17.3±13.2 |
| ***P* value** | 0.271027 | 0.582876 | 0.374694 |

HC, healthy control.

**Supplementary Table 3**  The data from SparCC network analysis between CD group and HC group

| **Genus 1** | **Genua 2** | **R value** | ***P* value** |
| --- | --- | --- | --- |
| *Clonostachys* | *Tausonia* | -0.65309 | ＜0.01 |
| *Clonostachys* | *Lophiostoma* | 0.878121 | ＜0.01 |
| *Clonostachys* | *Mucor* | 0.856629 | ＜0.01 |
| *Clonostachys* | *Fusarium* | 0.822864 | ＜0.01 |
| *Dipodascus* | *Cutaneotrichosporon* | 0.530485 | ＜0.01 |
| *Thermoascus* | *Cutaneotrichosporon* | 0.535289 | ＜0.01 |
| *Cutaneotrichosporon* | *Tausonia* | 0.510917 | ＜0.01 |
| *Tausonia* | *Lophiostoma* | -0.62826 | ＜0.01 |
| *Tausonia* | *Mucor* | -0.57554 | ＜0.01 |
| *Tausonia* | *Fusarium* | -0.55904 | ＜0.01 |
| *Lophiostoma* | *Mucor* | 0.850388 | ＜0.01 |
| *Lophiostoma* | *Fusarium* | 0.818527 | ＜0.01 |
| *Mucor* | *Fusarium* | 0.789676 | ＜0.01 |

In SparCC network analysis, based on the P value＜0.05, absolute value of R: 0.8-1.0 means extremely strong correlation, 0.6-0.8 means strong correlation; 0.4-0.6 means moderate correlation; 0.2-0.4 means weak correlation; 0-0.2 very weak correlation or no correlation.

**Supplementary Table 4**  *Exophiala dermatitidis* in CD-flare and CD-remission at different taxonomic levels

| **Taxonomic levels** | **Taxa** | **Relative abundance** | | **P value** |
| --- | --- | --- | --- | --- |
|  |  | **CD-flare** | **CD-remission** |  |
| OUT | OTU000005 | 0.464 | 15.769 | 0.007 |
| Species | *Exophiala dermatitidis* | 0.464 | 15.769 | 0.007 |
| Genus | *Exophiala* | 0.518 | 15.769 | 0.007 |
| Family | Herpotrichiellaceae | 0.543 | 15.788 | 0.007 |
| Order | Chaetothyriales | 0.91 | 15.827 | 0.009 |

CD-flare, CD at active stage (i.e. CDAI score ≥150); CD-remission, CD at remission stage (i.e. CDAI score <150).

**Supplementary Table 5**  Alpha diversity between CD-flare and CD-remission

| **Index** | **CD-flare** | **CD-remission** | **P value** |
| --- | --- | --- | --- |
| Observed species | 57.44±22.58 | 70.82±19.85 | 0.076 |
| PD-whole tree | 13.06±4.20 | 15.76±3.10 | 0.031 |
| Shannon | 2.93±1.36 | 3.45±1.45 | 0.310 |
| Simpson | 0.68±0.27 | 0.77±0.25 | 0.320 |
| Chao1 | 68.55±28.45 | 83.01±28.47 | 0.161 |
| ACE | 70.64±26.87 | 82.56±26.71 | 0.216 |
| Goods coverage | 0.1±0.0042 | 0.1±0.0005 | 0.313 |
| Pielou | 0.52±0.24 | 0.56±0.23 | 0.606 |

CD-flare, CD at active stage (i.e. CDAI score ≥150); CD-remission, CD at remission stage (i.e. CDAI score <150).

Supplementary Table 6 Baseline parameters between type B1 and type non-B1

|  | type B1 (n=20) | type non-B1 (n=25) | P value |
| --- | --- | --- | --- |
| Age, Mean±SD | 30.3±11.0 | 33.4±12.0 | 0.36 |
| Sex, n（male/female） | 18/2 | 16/9 | 0.08 |
| Flare/remission, n | 13/7 | 21/4 | 0.176 |
| Montreal classification, n |  |  |  |
| A1/A2/A3 | 1/15/4 | 1/20/4 | 0.92 |
| L1/L2/L3/l4 | 2/6/12/0 | 0/5/20/0 | 0.12 |
| B1/B2/B3 | 20/0/0 | 0/9/19 | <0.01 |
| Perianal complications, n | 12 | 13 | 0.60 |

Supplementary Table 7 Baseline parameters between CD patients with perianal lesions and without perianal lesions

|  | CD with perianal lesions (n=25) | CD without perianal lesions (n=20) | P value |
| --- | --- | --- | --- |
| Age, Mean±SD | 30.7±11.3 | 33.7±12.0 | 0.39 |
| Sex, n（male/female） | 20/5 | 14/6 | 0.44 |
| Flare/remission, n | 20/5 | 14/6 | 0.44 |
| Montreal classification, n |  |  |  |
| A1/A2/A3 | 2/19/4 | 0/16/4 | 0.29 |
| L1/L2/L3/l4 | 2/7/16/0 | 0/4/16/0 | 0.22 |
| B1/B2/B3 | 12/2/11 | 8/7/8 | 0.13 |
